# Machine Learning for Causal Inference in Biological Networks: Perspectives of This Challenge

**DOI:** 10.3389/fbinf.2021.746712

**Published:** 2021-09-22

**Authors:** Paola Lecca

**Affiliations:** Faculty of Computer Science, Free University of Bozen-Bolzano, Piazza Domenicani, Bolzano, Italy

**Keywords:** machine learning, deep learning, causality, inference, causal thinking, artificial intelligence, systems biology

## Abstract

Most machine learning-based methods predict outcomes rather than understanding causality. Machine learning methods have been proved to be efficient in finding correlations in data, but unskilful to determine causation. This issue severely limits the applicability of machine learning methods to infer the causal relationships between the entities of a biological network, and more in general of any dynamical system, such as medical intervention strategies and clinical outcomes system, that is representable as a network. From the perspective of those who want to use the results of network inference not only to understand the mechanisms underlying the dynamics, but also to understand how the network reacts to external stimuli (e. g. environmental factors, therapeutic treatments), tools that can understand the causal relationships between data are highly demanded. Given the increasing popularity of machine learning techniques in computational biology and the recent literature proposing the use of machine learning techniques for the inference of biological networks, we would like to present the challenges that mathematics and computer science research faces in generalising machine learning to an approach capable of understanding causal relationships, and the prospects that achieving this will open up for the medical application domains of systems biology, the main paradigm of which is precisely network biology at any physical scale.

## 1 Introduction

The availability of big data, the use of (deep) machine learning techniques to process them, and consequently the opportunity to access and/or perform high-performance computing are becoming of crucial importance for biology (Xu and Jackson, 2019), medicine and healthcare (Bates et al., 2020; Prosperi et al., 2020). Machine learning aims to develop computer algorithms that improve with experience and with the use of data. Nowadays, machine learning techniques are integrated with bioinformatic methods, as well as curated databases and biological networks, to enhance training and validation, identify the best interpretable features, and enable feature and model investigation (Auslander et al., 2021). This integration is not without its challenges and hurdles to overcome, but it is an endeavour that many researchers have decided to actively address given the tantalizing promise of machine learning to enable sophisticated analysis of complex biological systems.

In molecular biology, machine learning techniques are used for the analysis of genome sequencing data sets, including the annotation of sequence elements and epigenetic, proteomic or metabolomic data (Libbrecht and Noble, 2015). Xu et al. (Xu and Jackson, 2019) provide a brief but comprehensive overview of the main uses of machine learning in genomic applications. Machine learning has been used to predict the sequence specificities of DNA- and RNA-binding proteins, enhancers, and other regulatory regions (Libbrecht and Noble, 2015; Zou et al., 2018) on data generated by omics approach, such as DNase I hypersensitive sites (DNase-seq), formaldehyde-assisted isolation of regulatory elements with sequencing (FAIRE-seq), assay for transposase-accessible chromatin using sequencing (ATAC-seq), and self-transcribing active regulatory region sequencing (STARR-seq). Machine learning in molecular biology can be used also to build model to predict regulatory elements and non-coding variant effects *de novo* from a DNA sequence (Zou et al., 2018). Recently, machine learning approaches have been used in population and evolutionary genetics, to address questions such as the identification of regions under purifying selection or selective sweep. Moreover, machine learning approaches have been used to predict transcript abundance (Washburn et al., 2019), imputation of missing SNPs and DNA methylation states (Sun and Kardia, 2008; Angermueller et al., 2017), and to accurately calling genetic variants present in an individual genome from billions of short full-of-errors sequence reads (Poplin et al., 2018).

From applications in genetics and genomics, machine learning has quickly made the leap to applications in medicine, to address disease diagnosis, disease classification, and to precision medicine to assess disease risk, take preventive measures, make diagnoses and define personalized treatment. However, precision medicine, and more in general medicine, are not only about predicting risks and outcomes, but also about predicting clinical models. In this regard, Prosperi et al. (2020) point out that interventional clinical predictive models require the correct specification of cause and effect, and the calculation of alternative scenarios. The deduction of cause and effect relationships is generally done in experiments or with the help of data-driven approaches. Many questions in biomedical research can only be answered with observational studies. Unfortunately, however, unlike controlled experiments or well-planned, experimental randomized clinical trials, observational studies are subject to a number of potential problems that may jeopardize the reliability of their results. Factors that may bias the results of observational studies include selection bias resulting from the way study subjects are recruited or from differing rates of study attendance depending on the subjects’ cultural background, perception of the problem, age, or socioeconomic status, information bias, measurement error, and confounders (Hammer et al., 2009). Under these conditions and without a substantial *a priori* knowledge, causal inference is not feasible. On the other hand, data-driven prediction models - often implemented by machine learning algorithms - even assuming they are derived from an experiment in which several bias were minimised, should be used with great caution and their results should be subjected to critical review before interpretation. Although these methods are widely used to draw cause-effect relationships, attention must be paid to the fact that neither their parameters nor their predictions necessarily have a causal interpretation (Prosperi et al., 2020). Therefore, the belief that data-driven prediction models allow trustable decisions and efficient planning of interventions for precision medicine, and in general, for medicine, is doubtful.

The same problem can be found in molecular biology, where a computational method for inferring biological networks, such as gene regulatory, protein-protein, metabolic, and signalling networks, has been sought for years since the emergence of the paradigm of systems biology in the early 2000s. Critical reviews and comparative analyses of the various methods that have been proposed over the years can be found in various reviews and research papers over the last 10 years, some of which in these references (Veiga et al., 2010; Dongarra et al., 2011; Oates and Mukherjee, 2012; Omony, 2014; Chang et al., 2015; Zarayeneh et al., 2016; Angermueller et al., 2017; Liu et al., 2019; Lu et al., 2021), who principally present data integrative statistical methods and methods of network reconstruction and causal contextualization of reconstructed networks. The development of experiments which, especially in genetics and genomics, manage to collect large amounts of heterogeneous data, along with the today-real possibility of high-performing computers and cloud parallel architectures have made it possible to apply various machine (and deep) learning methods to the problem of deducing causality relationships in a biological network. Consequently, the scientific literature already proposes several articles in which machine (and deep) learning approaches are used and applied in the most appropriate ways for the specific context of investigation.

Very recent contributions can be found in (Yuan and Bar-Joseph, 2019a; Badsha and Fu, 2019; Kishan et al., 2019; Li F. et al., 2020; Muzio et al., 2020; Yazdani et al., 2020) and a primer on machine learning for life scientists, including an introduction to deep learning can be found in (Kishan et al., 2019). In particular, Camacho et al. (Kishan et al., 2019) discuss opportunities and challenges of the application of machine learning to network biology, and envisage the impact on disease biology, drug discovery, microbiome research, and synthetic biology. The reader can find a comprehensive review on the opportunities of intersection between machine learning and network biology in (Camacho et al., 2018). Machine learning approaches have been used more for network reconstruction and network inference than for *causal* inference, a task the latter of which has been partly attempted to be solved by informing inference algorithms with *a priori* knowledge about network nodes and edges. Nevertheless, the current literature provides a very solid and promising basis from which to implement machine learning approaches for causal discovery.

Despite the promising premises that these works hint at, it is the opinion of many experts in artificial intelligence that—using Schölkopf et al. (2021) words—if we compare what machine learning can do to what animals accomplish, we observe that the former is rather bad at some crucial feats where animals excel (Schölkopf, 2019; Schölkopf et al., 2021). These crucial operations include transfer to new problems, and any form of generalization that is not from one data point to the next one (sampled from the same distribution), but rather from one problem to the next one. Schölkopf et al. (2021) also note that this is not surprising, because machine learning often disregards information that animals use heavily, such as interventions in the world, domain shifts, and temporal structure. When designing machine learning algorithms, these factors are usually considered a nuisance and a source of noise, and are therefore not included. Yet these same factors are what would enable machine and deep learning methods to infer causal structures.

Finally, as we will discuss later in the paper, we recall that in any computational pipeline dedicated to causal inference, methods of identifying causal variables are of strategic importance. In this respect, there is already available a literature of advanced computational methods and advanced experimental technology. Noticeable findings that provide a solid basis on which to develop new methods for the identification of causal variables are, for example, studies aimed at the identification of cancer biomarkers as the study of Zhang et al. (2020a), who proposed a novel method for high-throughput identification of cancer biomarkers in human body fluids. Their use in research has increased greatly in current research, given the importance of biomarkers in defining the causal pathway of a disease (Mayeux, 2004). The method in (Zhang J. et al., 2020) integrates physicochemical properties and the weighted observed percentages and position-specific scoring matrices profiles to enhance their attributes reflecting the evolutionary conservation of the body fluid-related proteins. The least absolute selection and shrinkage operator feature selection are used to generate the optimal feature subset. By the same author (Zhang et al., 2019), is a paper on the collection of data also needed for the identification of the cancer biomarkers, and another paper introducing and discussing structure-trained predictors to predict protein-binding residues. Zhang J. et al. (2017a) presented also a method to detect bioluminescent proteins, that by virtue of their capability of emitting lights, can be served as biomarkers and easily detected in biomedical research, such as gene expression analysis and signal transduction pathways. Zhang and co-authors in (Zhang J. et al., 2017) collected a series of sequence-derived features known to be involved in the structure and function of bioluminescent proteins. These features include amino acid composition, dipeptide composition, sequence motifs and physicochemical properties. They found the combination of four types of features that outperforms any other combinations or individual features. To remove potential irrelevant or redundant features, they introduced Fisher Markov Selector together with Sequential Backward Selection strategy to select the optimal feature subsets.

In this paper, some possible scenarios for the development of current machine learning approaches towards machine learning approaches capable of inferring causal relationships between components of a biological system are presented. The paper does not pretend to cover all the issues involved in the effort to make machine learning capable of causality. Such an attempt is largely impossible at present. In fact, it is only in recent years that the use of machine learning techniques has become ubiquitous, and so it is only now that its limitations are beginning to be understood. However, we focus on a rather large and very topical and timely slice of current research on causal discovery in machine learning. i.e. the analysis of the issues and possible perspectives that machine learning has in structural causal model inference. Causal inference methods based on Pear’s (Pearl, 2010) and other theoretical work with counterfactuals and structural causal models (see a comprehensive summary of them in (Nogueira et al., 2021) and a seminal works in (Andrieu et al., 2003; Yin and Yao, 2016)) have recently paved the way for the improvement of machine learning models, especially in biology, biomedicine, and recently in epidemiology (Triantafillou et al., 2017; Glymour et al., 2019; Castro et al., 2020; Rivas-Barragan et al., 2020; Rose, 2020; Wilkinson et al., 2020; Raita et al., 2021), the increment transparency of model assumptions, and the help control for confounding variables, the understanding of counterfactual reasoning, and ultimately the increment of the understanding of the effect of training set selection bias, and the causal discovery (Piraino, 2018).

The paper outlines as follows: Section 2 introduces some basic concepts of machine learning and mathematics of structural models and presents the current state of the art and the possible imminent perspectives on the development of machine learning methods for the learning of causal graphs. In particular, the session proposes the perspective of a strong coupling between meta-modelling and meta-learning to overcome the current limitation of machine learning in performing causal discovery. Section 3, comment on popular machine learning algorithm who has been reformulated specifically for causal discovery, deepens the perspectives presented in Section 2, presents the possible difficulties and proposes modular meta-leaning upstream of meta-modelling as the next challenge and opportunity for the development of machine learning that wants to perform causal inference. Finally, Section 4 draws some conclusions. The perspectives presented and discussed in this paper are conceived with particular reference to biological networks of any scale (molecular, cellular, ecosystem), but remain valid also in other research areas where the problem of inferring causal relationships in a dynamical system is posed.

## 2 Machine Learning and Structural Causal Models

A machine learning algorithm is a computer program that is able to learn from data. Mitchell (Mitchell, 1997) provided this definition: ”A computer program P is said to learn from experience E with respect to some class of tasks T and performance P, if its performance at tasks in T, as measured by performance P, improves with experience E”. A variety of experiences E, tasks T, and performance measures P, can be used to build a machine learning algorithm (Goodfellow et al., 2016). Learning is the ability to perform the task, which are usually described in terms of how the machine learning should process an *example*. An example is a set of *features* that have been quantitatively measured from some object or event that we want to process with machine learning. An example is usually represented by a vector $x\in R^{n}$ , whose entries are features. Machine learning can perform many tasks, such as classification, regression, transcription, machine translation, anomaly detection, imputation of missing values, de-noising, probability density estimation (Goodfellow et al., 2016), but causal inference is still a challenge for it, because of its inability to implement a generalization from one problem to the next, rather than a generalization from a data point to the next (both sampled from the same distribution). Schölkopf et al. (2021) use an evocative term for the first form of generalization, that is “out-of-distribution” generalization. In order to understand the nature of the problem more fully, it is necessary to start with a formal description of a causal model, which we briefly outline in the following subsection adopting a notation similar to that of Schölkopf et al. (Schölkopf et al., 2021). We then present some perspectives for machine learning of a structural causal model.

### 2.1 Learning a Structural Causal Model: The Present and the Perspectives

Consider a set of variables *X*_1_, *…* , *X*_*n*_ associated with the vertices of a directed acyclic graph (DAG), and assume that each observable is

$$X_{i}=f_{i}\left(PA_{i};U_{i}\right).\;i=1,2,\ldots ,n$$ (1)

where *f*_*i*_ a deterministic function depending on *X*_*i*_’s parents in the graph (denoted by *PA*_*i*_) and noise random variable *U*_*i*_. Directed edges in the graph represent direct causation, since the parents are connected to *X*_*i*_ by directed edges and through (1) directly affect *X*_*i*_. The presence of a noise term make *X*_*i*_ itself a random variable, for which it is possible to define a conditional probability distribution *P* (*X*_*i*_|*PA*_*i*_) The noises *U*_1_, *…* , *U*_*n*_ are assumed to be jointly independent. If they were not, then by the Common Cause Principle there should be another variable that causes their dependence, and thus the model in (1) would not be causally sufficient.

The graph structure along with the joint independence of the noises implies the factorization of the joint probability *P* (*X*_1_, *X*_2_, *…* , *X*_*n*_) into causal conditionals as follows (Schölkopf et al., 2021)

$$P\left(X_{1},X_{2},\ldots ,X_{n}\right)=\Pi_{i=1}^{n}P\left(X_{i}|PA_{i}\right)=\Pi_{i=1}^{n}P\left(X_{i}|X_{i+1},X_{i+2},\ldots ,X_{n}\right)$$ (2)

The factorization decomposes the joint distribution into conditionals corresponding to the functions *f*_*i*_ in Eq. 1. These assignments can be interpreted as the causal relationships responsible for all statistical dependencies among the variables. A structural causal model allows a straightforward formalization of *interventions* as operations that modify the arguments of the function *f*_*i*_ in (1), e.g., changing *U*_*i*_, or changing the functional form of *f*_*i*_ itself. In particular, changing the form of *f*_*i*_ means changing the dependency of *X*_*i*_ on its parents (Spirtes et al., 1993; Neuberg, 2003; Schölkopf et al., 2021). It is worth to note that there is a substantial difference between the statistical model and the causal model. If we dispose of a causal model, by a learning process, causal reasoning allows us to draw conclusions on the effect of interventions, and potential outcomes. On the contrary, statistical models only allow to reason about the outcome of independent identically distributed experiments.

There are two fundamental elements in a structural causal model: the graph and the functions *f*_*i*_, and then the fundamental question to ask is whether it is possible to deduce from the data both the graph and the functions *f*_*i*_. The question does not have an easy answer considering the fact that the deducing the graph is in general depending on deducing the functions. A common method to infer the graph from data is performing conditional independence tests, i.e. testing whether two random variables *X* and *Y* are independent, given *Z*. The conditional independences are implied by the causal Markov condition stating that if G is a causal graph with vertex set *V* and *P* is a probability distribution over the vertices in *V* generated by the causal structure represented by G , G and *P* satisfy the Causal Markov Condition if and only if for every *X* ∈ *V*, *Y* is independent of *V*∖(Descendants(*X*) ∪Parents(*X*)) given Parents(*X*) (Hájek, 2011). The causal Markov condition holds regardless of the complexity of the functions appearing in an structural causal model (Schölkopf et al., 2021), and this is definitely an advantage of the condition independence tests. However, it is well-known that testing for conditional independence is a hard statistical problem, especially if *Z* is a continuous random variable (Shah and Peters, 2020). Another problem, highlighted by Schölkopf (Schölkopf et al., 2021) is that in the case of only two variables, the ternary concept of conditional independence is not applicable and the Markov condition thus has no non-trivial implications. A possible solution to both problems could be found by making assumptions on the function in the structural causal model. This is the route typically taken by machine learning methods. It is well known that in absence of assumptions on the functions *f*_*i*_ it not possible to generalize finite-sample data. To better explain these statements, let’s take a practical example.

A non-linear structural causal model is a generative process of the following form (Galanti et al., 2020):

$$\begin{array}{ccc} & & X∼P_{X}\\ & & E∼P_{E}\\ & & Y=g\left(f\left(X\right),U\right). \end{array}$$ (3)

The functions $g:R^{D_{f}+D_{U}}\to R^{D_{Y}}$ and $f:R^{D_{X}}\to R^{D_{f}}$ are non-linear unknown functions. Here, as in Eq. 1, *X* is the input random variable and *U* is the noise variable that is independent of *X*. We say that $X\in R^{D_{X}}$ causes $Y\in R^{D_{Y}}$ if they satisfy a generative process, such as Eq. 3.

For any joint distribution P(X,Y) of two random variables *X* and *Y*, there is a structural causal model, *Y* = *g* (*f*(*X*), *U*), such that, *X* ⊥ *U* and *f*, *g* are some measurable functions. Therefore, in general, deciding whether *X* causes *Y* or *vice versa* is ill-posed when only provided with samples from the joint distribution. However, for the one dimensional case (i.e., X,Y∈R ), under reasonable conditions, a representation

Y=g(f(X)+U) (4)

holds only in one direction (Zhang and Hyvärinen, 2010). The model for *Y* in Eq. 4 is an additive noise mode, and thus represents a restricted class of functions *f*, The restriction is considerable compared to the possibilities for *f* offered by model in Eq. 3. Assuming a noise additive model make it easier to learn functions from data, and can break the symmetry between cause and effect in the two-variable case. Schölkopf et al. (Schölkopf et al., 2021) report several literature references showing that given a distribution over *X*, *Y* generated by an additive noise model, one cannot fit an additive noise model in the opposite direction (i.e., with the roles of *X* and *Y* interchanged). The assumption can be justified when the function *f* depends weakly on *U* and *U* has a not too large variance.

Restriction of function classes is not the only strategy to simplify the causal structure inference. The so-called “distribution-shifts”, i.e. observing the systems in different environments and contexts may also help to infer causal structure. Different contexts can come for instance from different interventions and from heterogeneous non-stationary data (Zhang K. et al., 2017). In a very general scenario different contexts may require the execution of different tasks and, thus, prepare the ground for the meta-learning, i.e. learning algorithms that learn from the output of other learning algorithms. Meta-learning is derived from meta-modelling with which it shares many objectives. Both rely on meta-data, i.e. data that describe other data, to model a predefined class of problems. Both are used to define the output and input relationships and then may be used to identify the best model representing the behaviour of the data (Hartmann et al., 2019). Meta learning studies and approaches has started in 1980s and became popular after Schmidhuber (Schmidhuber, 2015) and Bengio’s works (Goodfellow et al., 2016). The interest in meta-learning accelerated especially after the massive use of deep learning and advanced machine learning algorithms. The increment of the difficulties to train these learning algorithms generated a stronger interest for meta-learning studies. Currently, the major application domains of meta-learning for machine learning in bioinformatics have been genomic survival studies in cancer research (Qiu et al., 2020), and the estimation of heterogeneous therapeutic treatment effects (Künzel et al., 2019; Rivas-Barragan et al., 2020). Let us therefore look in more detail at the methodology of meta-learning for identifying causal directions.

An interesting prospect for meta-learning is the development of methods for learning conditional probability functions rather than *f*_*i*_ functions. Learning probability models is a more general approach than learning dynamical functions, it requires less a priori knowledge, allows it to be questioned in the light of new knowledge, thanks to Bayes’ theorem, and incorporates a comparison of models and possible scenarios by comparing the likelihoods of the models themselves. To learn the joint distribution of two variables *X* and *Y* we can use their conditional distributions *p*_*X*|*Y*_ (*X*, *Y*) and *p*_*Y*|*X*_ (*X*, *Y*) alongside their marginal distributions *p*_*X*_ and *p*_*Y*_. In a Bayesian framework, we can write the following probabilities

$$\begin{array}{ccc} & & P_{X\to Y}\left(X,Y\right)=P_{X\to Y}\left(X\right)P_{X\to Y}\left(Y|X\right)\\ & & P_{Y\to X}\left(X,Y\right)=P_{Y\to X}\left(Y\right)P_{Y\to X}\left(X|Y\right) \end{array}$$

Let us assume that the true causal direction is *X* → *Y* and use the training distribution *p*_0_ (*x*, *y*) = *p*_0_(*x*)*p* (*y*|*x*), Thereafter, the distribution is changed to the transfer distribution *p*_1_ (*x*, *y*) = *p*_1_(*x*)*p* (*y*|*x*). According to a recent methodology proposed by Wong et al. (Wong and Damjakob, 2021) both networks, *X* → *Y* and *Y* → *X*, are meta-trained to the transfer distribution for *N* steps according to the following two step process:

1) The relationship between *X* and *Y* is learned using two models: one assumes *X* causes *Y*, the other the opposite causal direction;
2) the distribution of *X* is changed to a transfer distribution. Both models are retrained on the new data and the resulting likelihoods are recorded.

The Resulting Likelihood Are

$$L_{X\to Y}=\Pi_{n=1}^{N}P_{X\to Y,n}\left(x_{n},y_{x}\right),\;L_{Y\to X}=\Pi_{n=1}^{N}P_{Y\to X,n}\left(x_{n},y_{x}\right),\;$$ (5)

where *P*_*X*→*Y*,*n*_ denotes the trained Bayesian network after step *n*. The loss function is calculated as

$$R\left(\alpha \right)=\log\left[\sigma \left(\alpha \right)L_{X\to Y}+\left(1-\sigma \left(\alpha \right)\right)L_{Y\to X}\right]$$ (6)

where *α* is a structural parameter defining the causal direction and *σ*(⋅) is a sigmoid function. From Eq. 6, we can see that $\frac{\partial R}{\partial \alpha }>0$ if *L*_*X*→*Y*_ < *L*_*Y*→*X*_, i.e. if *P*_*X*→*Y*_ is better at explaining the transfer distribution than *P*_*Y*→*X*_. Bengio et al. (Bengio et al., 2020) showed that

$$E_{\text{Data\_transfer}}\left[\log\left(L_{X\to Y}\right)\right]>E_{\text{D\_transfer}}\left[\log\left(L_{Y\to X}\right.\right]$$ (7)

where E[⋅] denotes the expected value, and Data_transfer is the data drawn from the transfer distribution. Regarding Eq. 7, Wong et al. (Wong and Damjakob, 2021) observed that as the loss function modelling the correct direction (*X* → *Y*) only needs to update its estimate for the unconditional distribution *P*_*X*→*Y*_(*X*) from *p*_0_(*x*) to *p*_1_(*x*) while the reverse direction network (*Y* → *X*) needs to change both *P*_*Y*→*X*_(*Y*) and *P*_*Y*→*X*_ (*X*|*Y*), we indeed obtain that the loss function for the correct direction has a lower expected value. Then, in summary, the meta-training of the candidate networks, the estimation of loss function, its likelihoods and their expected values, provide a methodology to recover causal directions.

**FIGURE 1:**
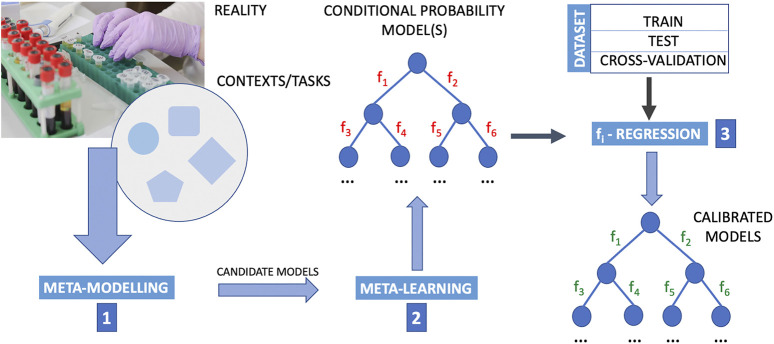
Outline of a computational procedure using upstream meta-modelling for the inference of causal structures. Meta-learning into a composite upside-down framework that includes the following phases in the following order. Step 1: meta-modelling first provide candidate models; step 2: meta-learning is designed to learn from data the conditional probability structure of these models where the structure and parameters of mathematical relations defining the interaction between nodes (i.e. the functions *f*_*i*_, *i* = 1, 2, *…* , *M* with *M* the number of arc in the probability graph) are then determined by regression methods (step 3). By employing meta-modelling upstream to meta-learning, and meta-learning it-self in place of a direct application of machine-learning, this pipeline extends a typical machine learning approach that generally poses the problem of structural causal discovery as a problem of learning the functions *f*_*i*_. In this pipeline, the determination of optimal *f*_*i*_ functions is posed as a regression problem once meta-modelling and meta-learning has identified wiring diagrams. The data are essential to the learning and regression procedure. The data are typically divided into train set, test set and cross-validation set. Cross-validation is a resampling procedure used to assess machine learning models on a limited data sample, and for the sake of simplicity in this figure is reported as a subset of the dataset. However, the procedure has a single parameter called *K* that refers to the number of groups that a given data sample is to be split into (for this reason for “cross-validation” it is usually meant K-fold cross-validation.). In K-fold cross validation we have multiple (K) train-test sets instead of 1, so that we train and test the model K-times. The purpose of doing this is that in a single train-test split, the test part of the data that we chose might be really easy to predict and the model will perform extremely well on it but not exactly so for the actual test sets. The image of the laboratory in this figure is part of the Pixabay free online pictures (https://pixabay.com/it/).

The deduction of causal directions with meta-learning is still a new research topic, but meta-learning is in perspective a very promising approach to develop machine learning methods capable of causal discovery. The greatest strength of meta-leaning, as we could see in the works of Wong and Damjakob (2021) and Bengio et al. (2020) here reported, lies in not considering assumptions on the data distribution itself, but rather on how it changes (e.g., when going from a training distribution to a transfer distribution, due some agent’s actions). However, there is a need to integrate meta-learning into a composite upside-down framework that includes the following phases in the following order: 1) meta-modelling. 2) meta-learning, and finally 3) testing of classes of candidate functions *f*_*i*_. Instead of posing the problem of learning the causal graph as a problem of determining *f*_*i*_ functions, one would pose it as an efficient process of learning for learning’s sake, and only at the end of this process, once a set of causal graphs has been obtained, e.g. according to schemes similar to Wong’s (Wong and Damjakob, 2021), one proceeds to the identification of the optimal *f*_*i*_ functions for each of these graphs. At this point the determination of the optimal *f*_*i*_ functions becomes a regression problem of the available experimental data. Figure 1 illustrates a scheme of this approach. Reducing the number of classes of functions in order to allow more nimble machine learning upstream of the whole learning procedure could create biases that are difficult to identify. Indeed, it could happen that the structure of the functions that is imposed at the beginning for simplifying purposes is such as to supply an incorrect causal model, precisely because it is too simplified or too abstract and therefore far from the physical reality of the task that one wants to learn. In these cases an evaluation of the goodness of the learning method based on measures of performance like the count of the false positives and false negatives and/or on other classic tests could not reveal this problem and in cases of very good performances it could even lead to think that the functions *f*_*i*_ are descriptive of the physical process that governs the interactions among the nodes of the graph.

In this new perspective the restriction of the classes of function is the results of a discrimination among the fitting classes of function, rather than an upstream simplification of the learning process. The starting point is instead the meta-modelling, i.e. construction and development of the frames, rules, constraints, and theories applicable for modelling a predefined class of problem. So, instead of restricting the class of possible functions, we start by considering a set of models for describing a class of problems, which in the language of machine learning derive from different contexts in turn obtained from different interventions. Meta-learning is then applied to learn the structural causal models outlined by meta-modelling. Preceding the machine learning phase by the meta-modelling phase could be a successful strategy to mimic the learning patterns of humans. Using a metaphor to explain machine learning from meta-models, we could say that people who know how to drive a car can most likely figure out how to drive a truck after a few instructions and a short demonstration. This may be a loosening and pursuable perspective, but it is not without its challenges. While meta-learning is evolving towards causal discovery meta-learning, popular classification algorithms, such as k-nearest neighbour (KNN), support vector machine (SVM), and random forests (RF), have been repurposed in the guise of causal KNN, causal SVM and causal RF as we see in the following sections. The KNN agorithm is the one whose mathematical specification has been most closely adapted to develop its own version for causal inference, and it is to this that we devote more space in the following. The other two approaches have instead been used more in combination with other techniques for causal inference in order to implement a computational pipeline for causal inference.

### 2.2 Causal K-Nearest-Neighbourhood

Zhou and Kosorok (2017) first and then Hitsch and Misra (Hitsch and Misra, 2018) introduced a way to use KNN to causal discovery. The method became rapidly popular and has been implemented in common statistics software libraries (see for example (Kricke and Peschenz, 2019)). In particular, in the application domain of precision medicine, the first proposed a causal k-nearest neighbour (CKNN) method to estimate the optimal treatment regime and the causal treatment effects within the nearest neighbourhood. The purpose was to tailor treatments to individual patients to maximize treatment benefit and reduce bad side-effects. Nevertheless, Zhou and Kosorok (2017) method is also applicable to biological networks, such as gene networks or protein-protein networks, when one wants to identify nodes or pathways affected by drug treatments or when one wants to design drug repurposing strategies, or drug combination prediction, and more in general to identify differences between networks of different patients. In this respect, we refer the reader to some significant recent studies such as (Feng et al., 2017; Cheng et al., 2019; Hasan et al., 2020; Lu et al., 2020; Adhami et al., 2021; Ruiz et al., 2021; Somolinos et al., 2021). In this context, the crucial step is the treatment selection rule, or optimal treatment regime. By “treatment regime” we mean a decision rule that assigns a treatment to a patient based on his or her clinical or medical characteristics. Similarly, for a biological network by “treatment regime” we mean a decision rule that assigns a therapeutic intervention to a network based on the state of the network or some of its pathways (e.g. altered pathways in disease state). In the next, we describe and comment on how the decision rule is constructed in a KNN method.

The KNN rule is a classification approach, where a subject is classified by a majority vote of its *k* neighbours. As noted by Zhou et al. (Zhou and Kosorok, 2017), the rationale of nearest neighbour rule is that close covariate vectors share similar properties more often than not. We briefly summarize causal KNN method, using a notation similar to that of Zhou and Kosorok (2017).

Consider a randomized clinical trial with *M* treatment arms. Let R∈R denote the observed clinical outcome, A∈A=1,.…,M denotes the treatment assignment received by the patient, and $X={\left(X_{1},\ldots ,X_{p}\right)}^{T}\in X\subset R^{p}$ , where *X* is compact,

Hldenotes the covariates, describing the feature of a node (e.g. the patient’s clinical covariates in a network patients-treatments, or gene expression in a gene network, or protein concentration in a protein-protein network). Let

$$\pi_{m}\left(x\right)=P\left(A=m|X=x\right)$$ (8)

denote the probability of being assigned treatment *m* for a node with covariates *x*. In the Zhou et al. framework this probability is assumed to be predefined in the design. Potential outcomes, denoted by R*(1), *…*, R*(*M*), are introduced and are defined as the outcomes that would be observed were a node to receive treatment 1, *…* , *M*, respectively. Very often in literature, we find two assumptions regarding the potential outcomes:

1) **Consistency assumption:** the potential outcomes and the observed outcomes agree, i.e.,

$$R=\Pi_{m=1}^{M}R^{*}\left(m\right)I\left(A=m\right)$$

where I (·) is the indicator function;

2) **No unmeasured confounders assumption:** conditional on covariates *X*, the potential outcomes {R*(1), *…*, R*(*M*)} are independent of the treatment assignment *A* that has been actually received.

Let’s make some remarks on these assumptions right away. We point out that it is often assumed that assumption two is valid in the case of randomised trials, but this belief is debatable. We consider it more prudent to state that in randomised trials the number of unmeasured confounders is reduced, but not completely eliminated and that, in any case, the influence on the predictive ability of the algorithm is not only given by the number of possible confounders, but also by their role in the system studied.

Mathematically, a treatment regime *d* is a function from covariates *X* to the treatment assignment *A*. For a treatment regime *d*, we can thus define its potential outcome Let

$$R^{*}\left(d\right)=\overset{M}{\underset{m=1}{\sum }}R^{*}\left(m\right)I\left(d\left(X\right)=m\right)$$ (9)

be the potential outcome of a treatment regime *d*. Let denote with $E\left(R^{*}\left(d\right)|X=x\right)$ the expected potential outcome under any regime *d*. An optimal regime *d*_opt_ is a regime that maximizes $E\left(R^{*}\left(d\right)\right)$ i.e.:

$$d_{\text{opt}}\left(x\right)={argmax}_{m=1,\ldots ,M}E\left(R^{*}\left(d\right)|X=x\right).$$ (10)

By the consistency assumption and non-unmeasured confounder assumption, we have that

$$E\left(R^{*}\left(d\right)|X=x\right)=E\left(R|X=x\;and\;A=m\right).$$

The causal nearest neighbour algorithm implements the following steps (i) to find a neighbourhood of *x* in X , (ii) to find an estimate $\hat{m}=E\left(R|X=x\;and\;A=m\right)$ for each arm in this neighbourhood, and (iii) to plug $\hat{m}$ into Eq. 10 to obtain the nearest neighbour estimate for the optimal treatment regime, i.e.

$$d_{\text{opt}}^{CKNN}\left(x\right)={argmax}_{m=1,\ldots ,M}\hat{m}.$$ (11)

$d_{\text{opt}}^{CKNN}\left(x\right)$ is called the causal k-nearest neighbour regime (Zhou and Kosorok, 2017).

We refer the reader to the works of Zhou and Kosorok (2017), Hitsch and Misra (Hitsch and Misra, 2018), (Kricke and Peschenz (2019) for the models used to calculate $\hat{m}$ and to the technical algorithmic details. What is important to note here is that the causal nearest neighbour regimes are calculated as local averaging, and *k* is a tuning parameter. It is required that *k* be small enough so that local changes of the distribution can be detected. On the other hand, *k* needs to be large enough so that averaging over the arm is effective. This parameter can be estimated by a cross validation procedure to balance the two requirements, provided we have a 1) sufficiently large sample and 2) sufficient computational resources to deal with large complex network. It is however well known that biological network inference is in many realistic situations an undetermined problem, since we size of the node covariate sample is small, whereas the size of the network is huge. However even assuming that computational costs can be managed and optimal covariate sample size can be obtained, still we have to deal with the problem that the potential outcomes of any node only depends on their nearest neighbours. The concept of neighbour is defined through the concept of distance, so ultimately the results of a CKNN in terms of both interpretation and reliability depend on the definition of distance we use. We point out that here by distance we do not mean the metrics e.g. Euclidean distance rather than Manhattan or Minkowski distance or others, but precisely the physical quantities and related processes we use in the definition of these metrics. For example, using the difference between the expression levels of genes in a gene network or protein concentrations in a protein-protein network might be insufficient for the purposes of causal inference, since cause-effect relations between nodes might not manifest themselves through a variation of this distance and might not manifest themselves only through appropriate behaviour of this distance.

### 2.3 Causal Random Forests

A random forest (RF) algorithm consists of many decision trees, i. e. a “forest” generated by the algorithm itself. The forest is trained through bootstrap aggregating. The RF algorithm establishes the outcome based on the predictions of the decision trees. It predicts by taking the mean of the output from various trees. Causal random forests (CRF) are recently proposed as a causal inference learning method that are an extension of Random Forests. In random forests, the data is repeatedly split in order to minimize prediction error of an outcome variable. Causal forests are built similarly, except that instead of minimizing prediction error, data are split in such a way to maximize the difference across groups in the relationship between an outcome variable and a “treatment” variable. Also in the context of CRF “treatment” is used in the broadest sense of the term. Causal forests simply estimate heterogeneity in a causal effect. In fact, the term causal referring to random forest can be misleading, as causal forests can reveal heterogeneity in a causal effect, but they do not by themselves make the effect causal. There have been interesting approaches to achieve this goal very recently, see for example the work of Li et al. (2020a) which developed a causal inference model combining Granger causality analysis and a random forest learning model. In the same vein, we find the works of Schmidt et al. (2020). and Tsai et al. (2020), all these devoted to identify cause-effect relationships in climatic phenomena, and at the present still not easily generalizable to the inference of biological networks of different kinds, due to the different physical nature of the climatic effects and the large variety of biological interactions. Nevertheless, on the same methodological line, there have also been important achievements in this direction in gene regulatory networks in the recent past, such as the development of a random forest algorithm for gene regulatory network inference by Petralia et al. (2015), Furqan and Siyal (2016), Deng et al. (2017), Huynh-Thu and Geurts (2018), Kimura et al. (2020), Zhang et al. (2020a), Cassan et al. (2021). The majority of the methods base on random forest for causal discovery in gene regulatory networks.

Most of these approaches implement upstream of the inference process the integration of large amounts of data of different natures that are indispensable for inferring causal relationships in structures as complex as biological networks. The complexity of a biological network, be it a gene regulatory network or a signalling network or a metabolic or biochemical network, lies in its size expressed by the number of nodes and the potential number of arcs and very often by the potential non-linear relationships between nodes that challenge the reliability and the accuracy of the regression techniques. The big amount of heterogeneous data would require the RF algorithm to generate a large quantity of trees to improve its efficiency. However, it is well known that the main limitation of random forest is that a large number of trees can make the algorithm too slow and ineffective for real-time predictions.

Furthermore, it is also well known that an RF algorithm cannot extrapolate. It can only calculate an average of previously observed labels. This means that when applied to a regression problem, a RF algorithm provide a range of predictions that is bound by the highest and lowest labels in the training data. This behaviour is regrettable when the training and prediction inputs differ in their range and/or distributions. This is called *covariate shift* and it is difficult for most models to handle (also to KNN) but especially for RF algorithms, because they only interpolate. The frequency with which the problem of covariate shift may be encountered is also very high when using heterogeneous biological data to aid causal inference in complex biological networks.

The current literature is promising regarding the applications of RF-based methods for causal inference in biological networks, but we believe that there are still many steps to be taken to overcome these limitations and to arrive at a mathematical model underlying RF angles that makes these approaches generalizable to networks other than gene regulatory networks, e.g. metabolic and biochemical networks.

### 2.4 Causal Support Vector Machine

Support vector machines (SVMs) appeared in the early nineties as optimal margin classifiers in the context of Vapnik’s statistical learning theory. The SVM algorithm aims to find a hyperplane in an *N*-dimensional space (where *N* is the number of features) that distinctly classifies the data points. SVMs can be used both for classification and regression tasks.

Moguerza and Muñoz (2006) highlights that an advantage of the support vector approach is that sparse solutions to classification and regression problems are usually obtained. i.e. only a few samples are involved in the determination of the classification or regression functions. This fact constitutes a facilitation of the application of SVMs to problems that involve a large amount of data, such as text processing and bioinformatics tasks. However, to the best of our knowledge, their evolution as a function of causal inference has not yet been developed, although in the literature we find some works in which SVMs are used in combination with other techniques in order to infer the structure of gene regulatory networks. In these regards, we report the work Gillani et al. (2014) who proposed CompareSVM a tool that can be used to infer gene regulatory network highly accurate for networks with less than 200 nodes. The tool employs SVM Gaussian kernel for biological datasets (knockout, knockdown, multifactorial and all). The authors state that for large network, choice of algorithm depends upon the type of biological condition. Interestingly they state that since there are variations in prediction accuracy in all inference methods, prediction should be limited for simple network. Furthermore they envisage that future work is needed for the development of semi-supervised methods capable of predicting targets of transcription factors which have no prior known targets.

Another study, representative of the works using SVMs in biological network inference, is the paper of Vert et al. (2007), who deal with inferring network edges in a supervised way from a set of high-confidence edges, possibly characterized by multiple, heterogeneous data sets (protein sequence, gene expression, etc.). In this setting the authors distinguish between two modes of inference: direct inference based upon similarities between nodes joined by an edge, and indirect inference based upon similarities between one pair of nodes and another pair of nodes. Theirs is a supervised approach for the direct case consisting of learning a distance metric. In this framework, a relaxation of the resulting convex optimization problem leads to the a SVM algorithm with a particular kernel for pairs, that is called “the metric learning pairwise kernel”. The proposed methods hold the promise of being used by most SVM implementations to solve problems of supervised classification and inference of pairwise relationships from heterogeneous data.

Finally, a recent work by Le Borgne et al. (2021) not specifically on biological networks, but on treatment-effect networks, found that SVM approach is competing with the most powerful recent methods, such as G-computation (Snowden et al., 2011) for small sample sizes with one hundred nodes when the relationships between the covariates and the outcome are complex. These findings, as well as the literature mentioned in this section, constitute important insights into the development of an efficient future causal version of SVMs.

## 3 The Challenges of the Modern Machine Learning

The two main challenges that machine learning algorithms have to face are:

• the need for large datasets for training and the high operational costs due to many trials/experiments during the training phase
• and the remarkable dependency of the algorithms results of the training data, and the risk of over-fitting.

These are the challenges of current machine learning approaches and should be distinguished from the challenges faced by users of these approaches, such as for instance:

• data quality control
• exclusion of irrelevant features.

Not in all areas of application of computational biology, we can count among the challenges of relevance to the user of machine learning, the collection of high-dimensional data samples, as this is not always experimentally feasible and could often be very expensive. For this reason, when dealing with biological networks, which can be not only gene networks (where indeed in the last decade the volume and heterogeneity of the data strongly continued to grow), but also biochemical networks, protein-protein interaction networks, metabolic network, and signalling networks (where the volume of experimental data is lower and the sample size is not always optimal), we prefer to indicate the ability to infer causal structures from a limited number of data as a challenge that computational procedures must try to win. The challenges for users of machine learning, listed here, are common to many computational approaches and are addressed by many textbooks and many innovative solutions presented in papers in the scientific literature. Given the large amount of literature on the subject and the fact that these challenges are not unique to machine learning algorithms, we do not address them in this work. Instead, in Section 3.1, we put into perspective two possible approaches to solving the problems concerning the large amount of training data and the risk of over-fitting, i.e., lacking predictive and abstractive capabilities. The first perspective concerns meta-modelling upstream of any learning procedure and in particular modular meta-modelling, while the second perspective concerns meta-learning and modular meta-learning. We believe that these two perspectives can contribute to overcoming the challenges posed to machine learning algorithms. In particular, the first perspective can be useful in overcoming the first challenge, while the second perspective can be useful in overcoming the second challenge, even if only the synergy between the methods proposed in the first and second perspective is much more effective in achieving both objectives in network biology. Finally, section 3.2 reports on one of the major problems that meta-learning methods also will have to solve, namely connecting causal variables to data, and the necessity to rely both on observational and interventional data.

### 3.1 Modular Meta-modelling for Modular meta-Learning

Meta-modelling goes in the direction of reducing the amount of needed data (for this purpose, recent meta-learning algorithm are also equipped with data-augmentation procedures (Ni et al., 2021)), and computational costs and times, but a further step in these directions can be taken by exploiting the modular structure of many physical systems. Biological systems, and specifically biological networks are known to have a modular organisation. to give an example, there several studies dealing with the mergence of modularity in gene networks (Rives and Galitski, 2003; Lorenz et al., 2011; Zhang and Zhang, 2013; Hütt, 2019; Serban, 2020) and in protein-protein network (Zhang et al., 2010). Modularity is a ubiquitous phenomenon in various biological systems, both in genotype and in phenotype. Biological modules, which consist of components relatively independent in functions as well as in morphologies, have facilitated daily performance of biological systems and their long time evolution in history. Indeed, modularity in biological networks is an emergent property evolved to be highly functional within uncertain environments while remaining remarkably adaptable. Modular organization is one of the main contributors to the robustness and evolvability of biological networks (Hintze and Adami, 2008). In order to understand the intimate connection between the modular organisation of a biological network and the improvement of meta-learning efficiency we start from the following considerations.

An artificial or natural system in a complex world is faced with limited resources. This concerns training data, i.e., we only have limited data for each task/domain, and thus need to find ways of pooling/re-using data (Schölkopf et al., 2021). It also concerns computational resources. A biological network has a limited size defined by the number of its nodes and arcs to solve the plethora of tasks in the daily life. Seen as a computational system that responds to stimuli and reconfigures its algorithms to changes in the surrounding environment, it is therefore forced to adapt its topology and consequently its computational performances to the environment conditions and the tasks it have to accomplish. The adaptability and the consequent evolvability of a biological network are made possible by the ability of the network to factor out variations of tasks and contexts. The modular structure of a biological network is the key of the mechanisms allowing to the network to factor out tasks and contexts.

Future machine learning models that aim to infer a biological network should be implemented to learn the modular structure of the biological network itself and also the crosstalks between the network modules. The latter capability in particular is the one that allows machine-learning for network inference to be carried out with less data and a limited number of computational resources. It is advisable that machine learning methods to learn the functional modules and their interactions mimic the way of learning of animal brain. In fact, modularity is the principle that provides a natural way of achieving compositionality and generalization. An example from Schölkopf’s work (Schölkopf et al., 2021) may help to better understand this statement. If, because of the variations of natural lighting the environment can appear in brightness conditions spanning several orders of magnitude, then visual processing algorithms in animal brain factor out these variations, so they do not need to build separate sets of object recognition algorithms for every lighting condition. Building different object recognizers would require considerable computational expenses and would involve the risk of not having sufficient computational resources within the physical dimensions of the brain.

**FIGURE 2:**
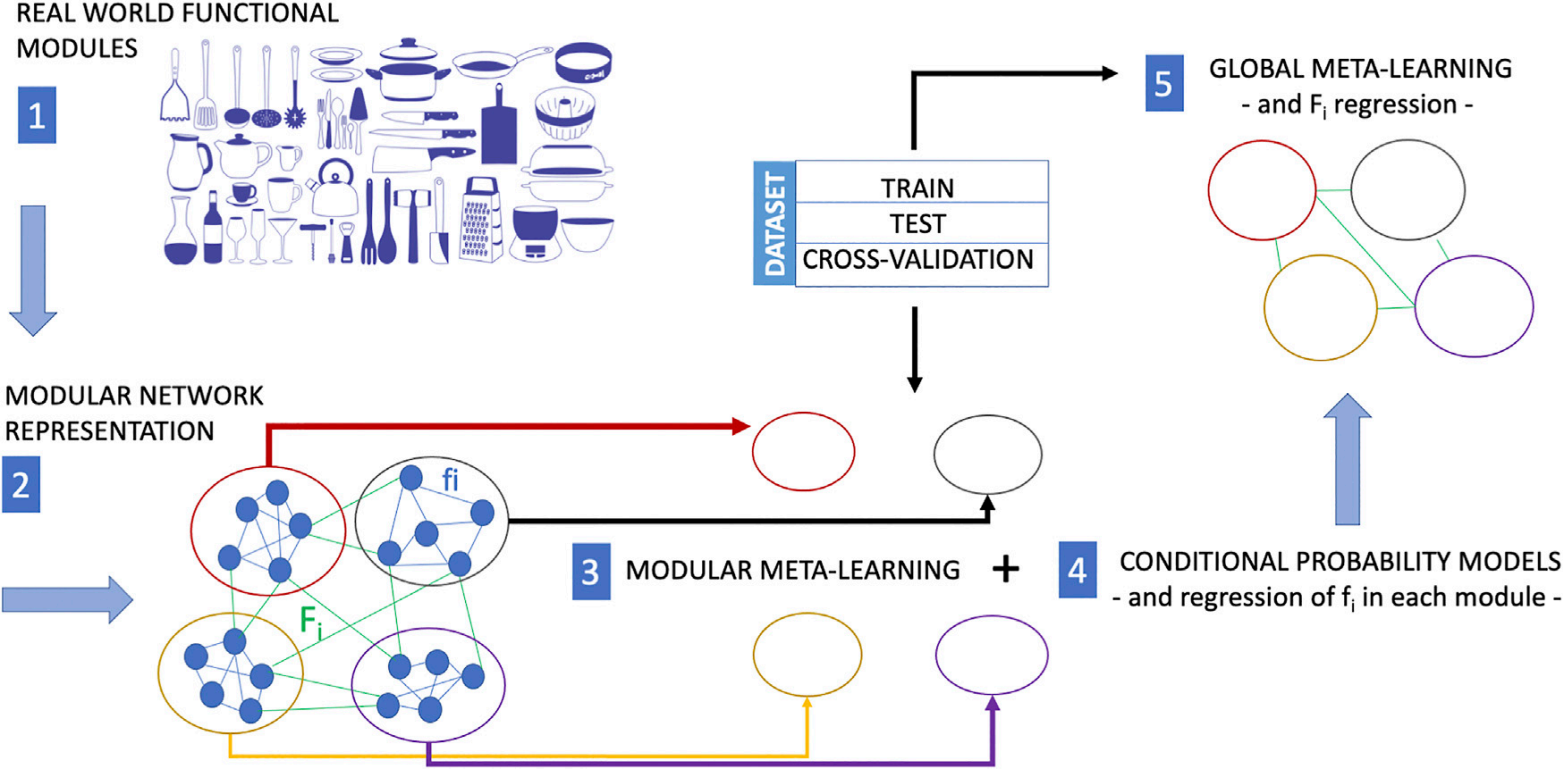
In many situations, training experience is very expensive. While meta-learning is a strategy to reduce the training-data requirements for a new task, modular meta-learning is a strategy to reduce or save computational resources. Modular meta-learning methods learn sets of network modules of a biological network. This learning scheme aims at mimicking the animal brain which is capable to factor out variations of a context or a task, and by virtue of this ability it does not need to implement different algorithms in separate anatomical regions to learn each single variation of a context or task. The functional modularity of a real system (here represented as a collection kitchen utensils with different functions) is first mapped into a modular network (each module of which performs a different function). The causal structure of processes within each module can be learned by modular meta-learning methods, and finally the causal structure of the interactions among network clusters is learned by meta-learning approaches including regression of the functions *f*_*i*_ internal to each modules (modular meta-learning) and then of the *F*_*i*_ representing the cross-talks between clusters (global meta-learning). The image of the kitchen utensils in this figure is part of the Pixabay free online pictures (https://pixabay.com/it/).

The modular structure and the interactions between the modules of a biological network, if reflected in an automatic learning procedure, are what allows artificial intelligence to factor out different variations of tasks and contexts, to save computational resources, and to require less training data. Paraphrasing a statement by Schölkopf (Schölkopf et al., 2021) regarding the components of an AI device, we could say that “if the world is indeed modular, in the sense that components/mechanisms of the world play roles across a range of environments, tasks, and settings, then it would be prudent for a machine learning approach to employ corresponding modules”. This also holds with regard to the capability of a machine learning model to implement causal discovery. *Modular* meta-learning for causal discovery could be the new research Frontier of the systems biology. Modular meta-learning would in fact allow learning sets of network modules (Alet et al., 2018) whose dynamic interactions and adaptive reconfiguration mechanisms could be learned subsequently. This perspective is depicted in Figure 2. A modular network maps the modular structure of the set of function of a real world system. Then, modular meta-learning approaches learn the causal structure of processes within each cluster of the network, and finally meta-learning approaches infer the causal structure of the network connecting the clusters. Regression procedures are applied at the end of modular meta-learning to determine the *f*_*i*_ functions and at the end of meta-learning to determine the *F*_*i*_ functions describing the dynamics of the crosstalks between network modules.

### 3.2 The Challenges of meta-Learning

In bioinformatics over the last 5 years, there have been studies using meta-learning approaches applied to the design of inferential systems (see for example Arredondo and Ormazábal (2015), who developed inference systems for the systematic classification of the best candidates for inclusion in bacterial metabolic pathway maps). However, these have mainly been inference procedures for deducing missing knowledge at the level of the network node rather than at the level of the arc and interaction mechanism, i.e. the causal relationship between nodes (Hill et al., 2016). Modular meta-learning in the schemes proposed in the previous section could be a step towards the evolution of machine learning algorithms for causal discovery. However, these schemes also face challenges. In order to function at their best, they must rely on observational and interventional data when causal variables are observed. It is indeed known that a central problem for AI and causality is, learning of high-level causal variables from low-level observations. A key research area at the intersection of modular meta-learning and causal modelling will be in the next future learning of causal variables and connecting them to the data and the task/contexts. Connecting causal variables to the data is at the moment an undetermined problem in machine learning and more specifically in modular meta-learning, as when a network is trained for sets of tasks, different high-level causal variables may be identified depending on the task. We mainly see this problem as the first next challenge to be faced by machine learning approaches for causal discovery, including more specialised approaches such as reinforcement learning and deep learning (Luo et al., 2020; Shen et al., 2020). The second major challenge will then be to identify indirect causal relationships, i.e. those relationships that would take place through latent mediating variables. To this end, current research focuses in particular on deep-learning methods, as testified by recent preliminary results published as a preprint (e. g.) (Yuan and Bar-Joseph, 2019b; Fan et al., 2021)), and envisaged by previous studies (e.g. (Ching et al., 2018; Jin et al., 2020)).

## 4 Conclusion

In the light of the above, the convergence of three research areas, namely experimental research guided by data acquisition protocols aimed at inferring causal relationships, machine learning, and graphical causal modelling, is becoming increasingly urgent. In the context of this convergence, the limitations of one area will have to be compensated for by the advances of another area. For example, experiments in biology or in the clinic, observational data can often provide observational data, due to experimental limitations or ethical codes. It is well known that causal inference from observational data is particularly difficult and its outputs are mostly unreliable. Observational data are affected by biases from confounding, selection and measurement, which can result in an underestimate or overestimate of the effect of interest (Hammerton and Munafò, 2021). In this case, it is expected that in the near future machine learning methods will be able to identify high-level causal variables from low-level data, and causal modelling approaches will be able to output accurate causal relationships. Meta-modelling and meta-learning are two approaches conceived in the logic of “doing more with less” (Hammerton and Munafò, 2021). This is why they want to imitate the animal brain in learning causality, focusing on its ability to generalise to different contexts, and thus using a smaller amount of training data and a limited amount of computational resources. However, we should not forget that this advantage can come at a cost. Machine learning is a computational process. To that end, it is strongly tied to computational power and hardware supporting it. Hardware shapes the methods used in the design and development of machine learning models. Characteristics such as the power consumption of chips also define *where* and *how* machine learning can be used in the real world. At present, it is not yet possible to have a clear idea of the components and hardware architecture that computational schemes such as those shown in Figure 1 and Figure 2 might require. This is therefore another line of research that needs to be pursued, not only to understand its impact on artificial intelligence, but also on the systems biology and medicine, and, more importantly, on the community.

## Data Availability

The original contributions presented in the study are included in the article, further inquiries can be directed to the corresponding author.
