# Corrigendum: Machine Learning for Causal Inference in Biological Networks: Perspectives of This Challenge

**DOI:** 10.3389/fbinf.2022.888273

**Published:** 2022-04-06

**Authors:** Paola Lecca

**Affiliations:** Faculty of Computer Science, Free University of Bozen-Bolzano, Bolzano, Italy

**Keywords:** machine learning, deep learning, causality, inference, causal thinking, artificial intelligence, systems biology

## Figure 1 legend

In the original article, there were mistakes in the legend for Figure 1 as published.

**FIGURE 1:**
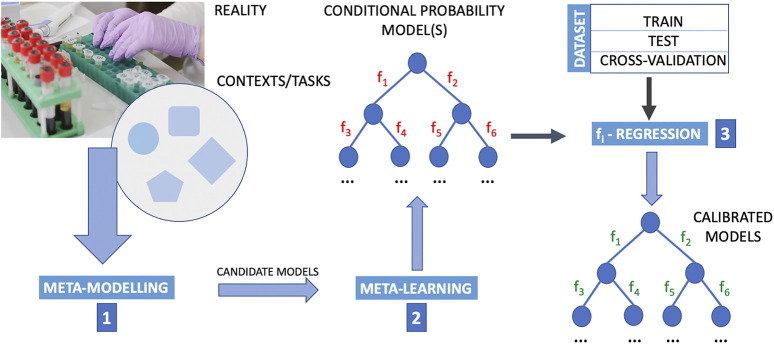
Outline of a computational procedure using upstream meta-modelling for the inference of causal structures. Meta-learning into a composite upside-down framework that includes the following phases in the following order. Step 1: meta-modelling first provide candidate models; step 2: meta-learning is designed to learn from data the conditional probability structure of these models where the structure and parameters of mathematical relations defining the interaction between nodes (i.e., the functions *f*_*i*_, *i* = 1, 2, *…* , *M* with *M* the number of arcs in the probability graph) are then determined by regression methods (step 3). By employing meta-modelling upstream to meta-learning, and meta-learning it-self in place of a direct application of machine-learning, this pipeline extends a typical machine learning approach that generally poses the problem of structural causal discovery as a problem of learning the functions *f*_*i*_. In this pipeline, the determination of optimal *f*_*i*_ functions is posed as a regression problem once meta-modelling and meta-learning has identified wiring diagrams. The data are essential to the learning and regression procedure. The data are typically divided into train set, test set and cross-validation set. Cross-validation is a resampling procedure used to assess machine learning models on a limited data sample, and for the sake of simplicity in this figure is reported as a subset of the dataset. However, the procedure has a single parameter called *K* that refers to the number of groups that a given data sample is to be split into (for this reason for “cross-validation” it is usually meant K-fold cross-validation.). In K-fold cross validation we have multiple (K) train-test sets instead of 1, so that we train and test the model K-times. The purpose of doing this is that in a single train-test split, the test part of the data that we chose might be really easy to predict and the model will perform extremely well on it but not exactly so for the actual test sets. The image of the laboratory in this figure is part of the Pixabay free online pictures (https://pixabay.com/it/).

Mistakes which were made:

1) Lowercase letter after a full-stop: “…causal structures. meta-learning” instead of “…causal structures. Meta-learning”
2) “meta-moelling” instead of “meta-modelling”
3) “simplicitly” instead of “simplicity.”

The original article has been updated

## Figure 2 legend

In the original article, there was a mistake in the legend for Figure 2 as published. Mistakes which was made: the sentence “Outline of a computational procedure using upstream *modular* meta-modelling for the inference of causal structures.” has to be removed since it is has been copied by mistake from the caption of Figure 1. The correct legend appears below.

**FIGURE 2:**
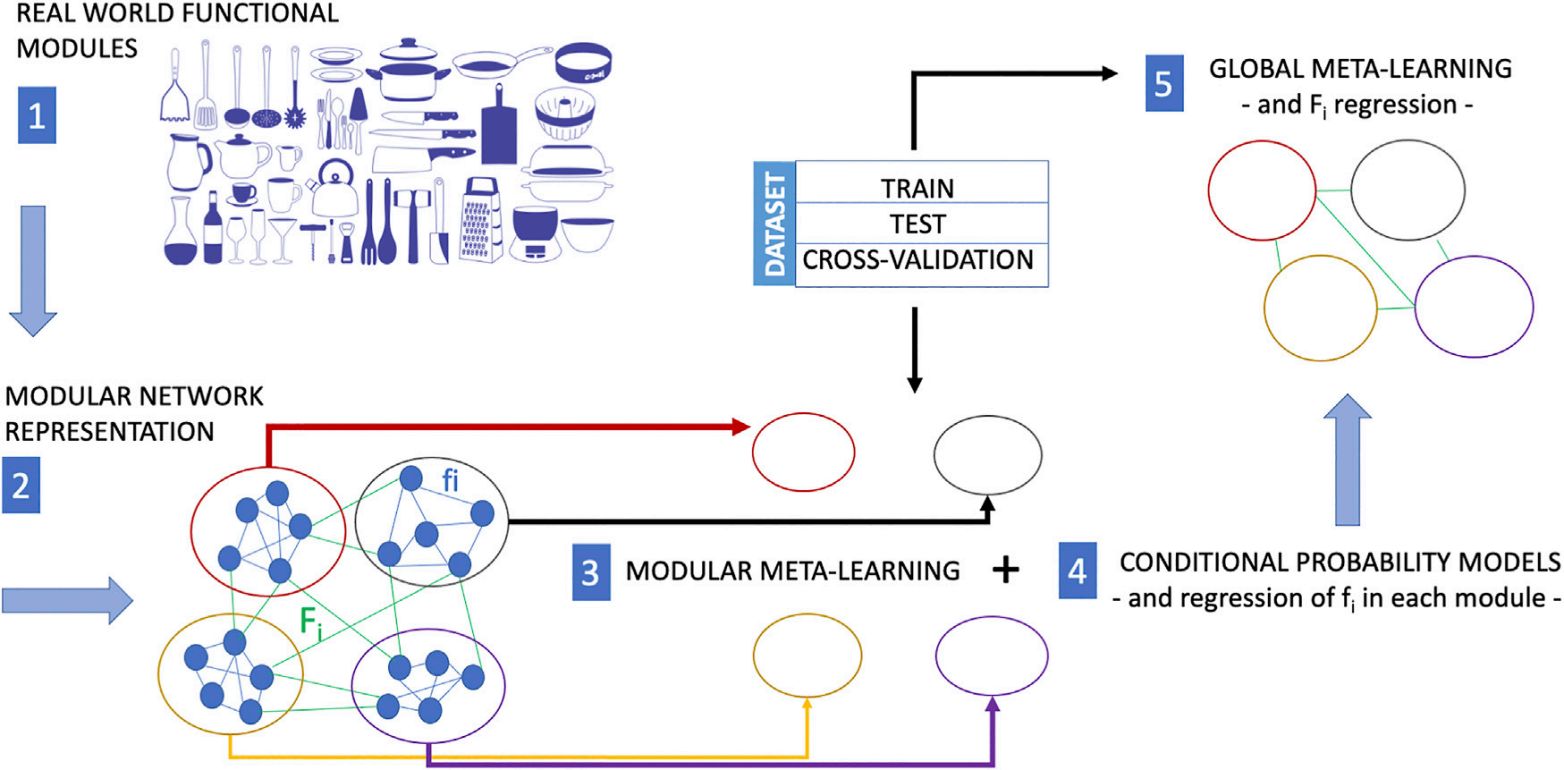
In many situations, training experience is very expensive. While meta-learning is a strategy to reduce the training-data requirements for a new task, modular meta-learning is a strategy to reduce or save computational resources. Modular meta-learning methods learn sets of network modules of a biological network. This learning scheme aims at mimicking the animal brain which is capable to factor out variations of a context or a task, and by virtue of this ability it does not need to implement different algorithms in separate anatomical regions to learn each single variation of a context or task. The functional modularity of a real system (here represented as a collection kitchen utensils with different functions) is first mapped into a modular network (each module of which performs a different function). The causal structure of processes within each module can be learned by modular meta-learning methods, and finally the causal structure of the interactions among network clusters is learned by meta-learning approaches including regression of the functions *f*_*i*_ internal to each modules (modular meta-learning) and then of the *F*_*i*_ representing the cross-talks between clusters (global meta-learning). The image of the kitchen utensils in this figure is part of the Pixabay free online pictures (https://pixabay.com/it/).

The original article has been updated

## Text Correction

In the original article, there were some errors.

1) Mistake that was made: verb in the infinitive instead of in the gerund mode, specifically "represent" instead of "representing".

A correction has been made to **Section 2**, **Sub-section 2.1** on page 5 in the middle of the second column, as follows:

“Both are used to define the output and input relationships and then may be used to identify the best model representing the behaviour of the data (Hartmann et al., 2019).”

2) Mistake that was made: name in the plural instead of the singular, specifically “problems” instead of “problem”.

A correction has been made to **Section 2**, **Sub-section 2.2** on page 8 at the beginning of the first column. The correct sentence is

“It is however well known that biological network inference is in many realistic situations an undetermined problem, since the size of the node covariate sample is small, whereas the size of the network is huge.”

3) Mistake that was made: repeated sentence

A correction has been made to **Section 2,** at the end of **Sub-section 2.2**. One instance of the sentence below was removed.

“For example, using the difference between the expression levels of genes in a gene network or protein concentrations in a protein-protein network might be insufficient for the purposes of causal inference, since cause-effect relations between nodes might not manifest themselves through a variation of this distance and might not manifest themselves only through appropriate behaviour of this distance.”

4) Mistake that was made: typos and singular/plural use errors. “ad G-computations” instead “as G-computation”; “This findings” instead of “These findings”; “…in this sections … ” instead of “…in this section … ”

A correction has been made to **Section 2**, at the end of **Sub-section 2.4**. The correct sentence appears below.

“Finally, a recent work by Le Borgne et al. (2021) not specifically on biological networks, but on treatment-effect networks, found that SVM approach is competing with the most powerful recent methods, such as G-computation (Snowden et al., 2011) for small sample sizes with one hundred nodes when the relationships between the covariates and the outcome are complex. These findings, as well as the literature mentioned in this section, constitute important insights into the development of an efficient future causal version of SVMs.”

5) Mistakes that were made: singular instead of a plural. i.e., “challenge” instead of “challenges,” and a full-stop instead of “:”

A correction has been made to **Section 3**, in the first sentence. The correct sentence is

“The two main challenges that machine learning algorithms have to face are: …”

The authors apologize for these errors and state that this does not change the scientific conclusions of the article in any way.

